# Prediction of heart disease and classifiers’ sensitivity analysis

**DOI:** 10.1186/s12859-020-03626-y

**Published:** 2020-07-02

**Authors:** Khaled Mohamad Almustafa

**Affiliations:** Department of Information Systems, College of Computer and Information Systems, Prince Sultan University, Riyadh, Kingdom of Saudi Arabia

**Keywords:** Heart disease (HD), Prediction, Classification, K-nearest neighbor, Support vector machine (SVM), Decision tree J48, Feature selection, Sensitivity analysis

## Abstract

**Background:**

Heart disease (HD) is one of the most common diseases nowadays, and an early diagnosis of such a disease is a crucial task for many health care providers to prevent their patients for such a disease and to save lives. In this paper, a comparative analysis of different classifiers was performed for the classification of the Heart Disease dataset in order to correctly classify and or predict HD cases with minimal attributes. The set contains 76 attributes including the class attribute, for 1025 patients collected from Cleveland, Hungary, Switzerland, and Long Beach, but in this paper, only a subset of 14 attributes are used, and each attribute has a given set value. The algorithms used K- Nearest Neighbor (K-NN), Naive Bayes, Decision tree J48, JRip, SVM, Adaboost, Stochastic Gradient Decent (SGD) and Decision Table (DT) classifiers to show the performance of the selected classifications algorithms to best classify, and or predict, the HD cases.

**Results:**

It was shown that using different classification algorithms for the classification of the HD dataset gives very promising results in term of the classification accuracy for the K-NN (K = 1), Decision tree J48 and JRip classifiers with accuracy of classification of 99.7073, 98.0488 and 97.2683% respectively. A feature extraction method was performed using Classifier Subset Evaluator on the HD dataset, and results show enhanced performance in term of the classification accuracy for K-NN (*N* = 1) and Decision Table classifiers to 100 and 93.8537% respectively after using the selected features by only applying a combination of up to 4 attributes instead of 13 attributes for the predication of the HD cases.

**Conclusion:**

Different classifiers were used and compared to classify the HD dataset, and we concluded the benefit of having a reliable feature selection method for HD disease prediction with using minimal number of attributes instead of having to consider all available ones.

## Backgrounds

Heart disease (HD) is one of the most common diseases nowadays, due to number of contributing factors, such as high blood pressure, diabetes, cholesterol fluctuation, exhaustion and many others. An early diagnosis of such disease has been sought for many years, and many data analytics tools have been applied to help health care providers to identify some of the early signs of HD. Many tests can be performed on potential patients to take the extra precautions measures to reduce the effect of having such a disease [1], and reliable methods to predict early stages of HD, such as the methods proposed in this paper, can be a crucial task for saving lives. Number of Machine Learning (ML) algorithms, such as, Naïve Bayes, Stochastic Gradient Descents (SGD), Support Vector Machine (SVM), K- Nearest Neighbor (K-NN), Adaboost, JRip, Decision tree J48, and others were applied for the purpose of classification and prediction of HD dataset, and many promising results were presented in the literature [2].

Due to the complex nature of the HD, suggested tests, which has to be prioritized [3], and proposed techniques have to be selected carefully, where authors worked on accurately and efficiently predict heart-related hospitalizations based on the available patient-specific medical history, and five machine learning algorithms, namely SVM, AdaBoost, logistic regression, a naïve Bayes event classifier where used, and results showed were consistent for all used classifiers for achievable prediction accuracy with a detection rate of 82%. Authors in [4] proposed an algorithm to predict the existence of heart disease using Back Propagation MLP (Multilayer Perceptron) of Artificial Nerual Network on a given HD dataset classifications, and ML algorithms, mainly neural networks for the predication of HD cases was used in [5], where authors proposed to develop an application which can predict the vulnerability of a heart disease given basic symptoms like age, sex, pulse rate, and neural networks showed the most accurate and reliable algorithm for the proposed system. A data mining model has been developed [6] using Random Forest classifier to improve the prediction accuracy and to investigate various events related to Heart Disease, and experimental results showed that classification using Random Forest Classification algorithm can be successfully used in predicting the events and risk factors related to HD. A hybrid method for HD prediction was proposed in [7] based on risk factors, where authors presented different data mining and neural network classification technologies used in predicting the risk of occurring heart diseases, and it was shown that classifying the risk level of a person using techniques like K-Nearest Neighbor Algorithm, Decision Trees, Genetic algorithm, Naïve Bayes is high when using more attributes and combinations of above techniques. Computer aided decision support system was presented in [8], and showed a reduction in prediction time for HD dataset, and supervised learning techniques for HD dataset prediction was proposed in [9]. Authors in [10] introduced particle swarm optimization to generate evolutionary values for HD, also good classification accuracy for HD dataset was presented in [11], in the form of a comparative analysis of different machine learning algorithms for diagnosis of heart disease as a survey paper, and it showed the suitability of machine learning algorithms and tools to be used for the analysis of HD, and decision-making process accordingly. An analysis system and follow up for HD dataset detection was proposed in [12] by building a mobile application capable of real-time diagnosis and monitoring of patients with Coronary Artery Disease (CAD) with a classification performance accuracy of more than 85% with the cross-validation test. Authors in [13] used Naïve Bayes classification algorithm to diagnose HD cases and proposing a Heart Diseases Prediction System (HDPS) by analyzing some of the parameters of the algorithm. Prediction of HD disease using K-mean clustering algorithm was shown in [14], where authors proposed an efficient hybrid algorithmic approach for heart disease prediction by considering 14 attributes out of 74 attributes of UCI Heart Disease Data Set, as the one used in our paper, and taking age, weight, gender, blood pressure and cholesterol rate into consideration as prediction parameters. A novel framework using non-linearity was proposed by [15, 16] to examine the heart rate variability, and different classification algorithms were presented. In [17], authors proposed a reasonable model for HD risk level prediction using classifications decisions rules. Algorithm for HD dataset classification using Neural Networks (NN) was proposed in [18] using 13 medical attributes for heart disease predictions with experimental results showing a good performance of the proposed algorithm compared to other prediction algorithms. Artificial Neural Network (ANN) classifiers were shown in [19] for the prediction of the HD dataset using back propagation algorithm for training the network and by using 13 clinical features as input and predicting absence or presence of heart disease with accuracy of 95%. Multiple predecessor techniques using ANN and other machine learning techniques were also presented in [20] by using UCI Laboratory data, and applying discovery pattern algorithms including Decision tree, Neural Networks, Rough Set, SVM, Naive Bayes, and compare their accuracy and prediction, and achieving an F-measure of 86.8%. Artificial Neural Network (ANN) algorithm for the classification of Carotid artery stenting (CAS) disease was proposed in [21], the data of 317 patients from Taiwan Nation Health Insurance Research Database (NHIRD) was used to train and test the constructed ANN model with an input features contain 13 clinical risk factors and the output is the occurrence of the Major Adverse Cardiovascular Events (MACE). The performance of their model showed 89.4% sensitivity, and an accuracy of 82.5%. Classification of HD dataset using voting techniques in classification and prediction was proposed in [22]. Hybrid methods for diminution reduction was presented in [23], where authors presented a methodology which uses the results of medical tests as input, extracts a reduced dimensional feature subset by using Probabilistic Principal Component Analysis (PPCA), and provides diagnosis of heart disease using UCI dataset. The proposed technique achieved an average accuracy of 86.43% over the used dataset. In [24] a classification model for coronary Heart Disease was proposed by utilizing Support Vector Machine (SVM) as well as Artificial Neural Network (ANN), and introducing a medical choice backing framework for coronary illness characterization in a sane, purpose, precise and fast manner using the Cleveland Heart Database and Statlog Database taken from UCI Machine learning dataset, and presenting a good results in classification accuracy and training time. Authors in [25] introduced a prediction system for heart disease using multilayer perceptron neural network, the NN in the proposed system accepts 13 clinical features as input and it is trained using back-propagation algorithm to predict the presence or absence of heart disease in the patient with a high accuracy of 98% for prediction.

More recently, Authors in [26] used Nasarian Coronary Artery Disease (CAD) dataset, in which work place and environmental features are also considered, in addition to other clinical features and results showed the proposed feature selection method has yielded the classification accuracy of 81.23% with SMOTE and XGBoost classifier. Authors in [27] compared the previous studies carried out by various researchers based on knowledge acquisition and presentation of expert system for diagnosis of coronary artery disease and presented their weaknesses. In [28], Authors used the extension of the Z-Alizadeh Sani dataset, containing 303 records with 54 features, and a new feature selection algorithm was proposed discretization of data to handle the uncertainty in CAD prediction. Discrete wavelet transform (DWT) coupled with novel 1-dimensional hexadecimal local pattern (1D-HLP) technique for the automated detection of arrhythmia detection was employed in [29], and a classification accuracy of 95.0% in classifying 17 arrhythmia classes using MIT-BIH Arrhythmia ECG dataset was obtained. An automated heartbeat classification based on nonlinear morphological features and a voting scheme suitable for rare heartbeat morphologies was presented in [30], their algorithm tested on MIT-BIH database, and the simulation results showed the superiority of their proposed method, especially in predicting minority groups with 90.4 and 100% classification. An approach for discovering classification rules of Coronary artery disease (CAD) was proposed by [31], and it was based on the real-world CAD data set and aims at the detection of this disease by producing the accurate and effective rules, and results showed that the proposed approach has the ability to produce effective rules with highest accuracy for the detection of CAD. An accurate detection of Coronary artery disease (CAD) for Iranian patients was applied in [32] using traditional machine learning algorithms, and to improve the performance of these algorithms, a data preprocessing with normalization was carried out with an accuracy of 93.08% for N2Genetic-nuSVM algorithm. The spectral power density for heart disease was estimated in [33] based on 744 segments of ECG signal from the MIT-BIH Arrhythmia database, and long-duration ECG signal segments was used, and the developed system achieved a recognition sensitivity of 94.62% and an accuracy of 99.37% in detecting 17 arrhythmia ECG classes.

In this paper, we will present *a comparative analysis* of the HD dataset classification using different classification algorithms, in which these classifiers are most used for similar bioinformatics related projects for datasets classifications. These classifiers were used with cross validation, with 10 folds method, to evaluate the performance of these classifiers to classify the available HD dataset, then we will study the performance of the Naïve Bays classifier using different training set instead of the cross validation method using 10 folds classification. *A sensitivity analysis*, as a contribution to this paper, will be applied to investigate the performance of the Decision tree J48 classifier based on the changes of its prune confidence factor parameter as an extra measure for the performance of this classifier, and to investigate a possible better classification with changes to such parameter. At last, we will apply *Feature Extraction* method, as a main contribution for HD prediction, using Classifier Subset Evaluator to estimate the accuracy of these subsets for all used classifiers on the HD dataset in order to evaluate the classification performance after selecting the relevant attributes per classification algorithm, so a better HD cases can be predicted with minimal number of attributes using the prediction algorithms suggested in this paper.

This paper is organized as follows. Section 2 contains the results, section 3 the discussion, methods are presented in section 4, and sections 5 and 6 present the conclusion and future work.

### Introduction and preparation of the heart disease dataset

The presented dataset in this paper is collected from [34], which is a summarized version of the dataset available in [35]. The set contains 76 attributes including the class attribute, for 1025 patients collected from Cleveland, Hungary, Switzerland, and Long Beach, but in this paper, only a subset of 14 attributes are used, mainly, age, sex, chest pain type, resting blood pressure, serum cholesterol, fasting blood sugar, resting electrocardiographic results, maximum heart rate achieved, exercise induced angina, old peak, the slope of the peak exercise ST segment, number of major vessels flourosopy and defect along with the class attribute, and each attribute has a set value, in term of its class value, similar to many published paper using same dataset for strongly imbalanced data as presented in [35]. The dataset attributes and their values are presented in Table 1.

**Table 1:** Heart Disease Dataset’s Attributes

Attribute	Code given	Note	Attributes Values
1. age	Age	in years	Numeric
2. sex	Sex	1 = male; 0 = female	Binary
3. chest pain type	level of pain	0,1,2,3	4 values
4. resting blood pressure	trestbps	in mm Hg	Numeric
5. serum cholesterol	cholesterol	in mg/dl	Numeric
6. fasting blood sugar	fbs	> 120 mg/dl	Numeric
7. resting electrocardiographic results	restecg	0,1,2	3 values
8. maximum heart rate achieved	thalach	71–202	Numeric
9. exercise induced angina	exang	0,1	Binary
10. oldpeak = ST	oldpeak	depression	Numeric
11. the slope of the peak exercise ST segment	slope	0,1,2	3 values
12. number of major vessels flourosopy	ca	0,1,2,3	4 values
13defect: normal;fixed;reversible; non-reversible	thal	0,1,2,3	4 values
14. class	target	0,1	Binary

**Fig. 1:**
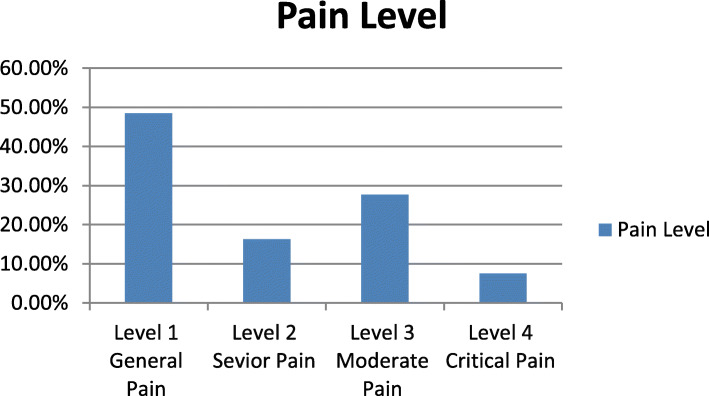
Distribution of the Chest Pain level Between Participants

Figure 1 shows the distribution of the of the chest pain level between participants, and we can see that most of the patients diagnosed with level 1, general pain.

## Results

This section gives the results of the methods used for classification of the HD dataset using different classifiers by using cross validation method with 10 folds. Sensitivity Analysis of Decision tree J48 classifier in term of its pruning confidence factor parameter is performed to see the changes of the classifier’s performance in term of the changes of this parameter. Then a classifier subset evaluator was used for feature selections of the HD dataset’s features to produce the proposed prediction models for different classifiers.

### Using different classifiers

The following section describes the results obtained using different classifiers on the heart disease dataset with cross validation method with 10 folds using WEKA software solution, version 3.8.4. The University of Waikato, Hamilton, New Zeeland, using a window 10 pro, Intil® core (TM) *i5* CPU, 4GB RM, 64-bit Operating System. Parameters for these classifiers are the default parameters by the software, unless otherwise specified as per the sensitivity analysis section of this paper.

### Parameter’s sensitivity

We will present some parameters sensitivity for Decision tree J48 classifier and change its pruning confidence factor parameter, where smaller pruning value would give more pruning, and we will study the accuracy performance, kappa statistic, MAE and RAE performance of the Decision tree J48 classifier due to these changes. Decision tree J48 was used for the sensitivity analysis, because it had the max accuracy percentage out of all other classifiers. Also, the training sample size for Naive Bay classifier will be used as a sensitivity parameter, by changing its training set size and observe the changes in its classification accuracy with respect to the portion of the training samples with respect to the total samples. Naïve Bay was selected as an example of low accuracy rate classifier, ad to see the changes of its performance in term of the changes of the training sample size. Regarding the sensitivity analysis, parameter start with the default value of the parameter, then it was changed accordingly to study the changes of the classifier performance in term of these parameters.

#### Decision tree J48 pruning confidence factor (PCF)

Pruning is one of the characteristics associated with the Decision tree J48 classifier, and Pruning Confidence Factor (PCF) is one of its parameters, and less value of such parameter means more pruning, and our used value for the classifiers comparison in the previous section was PCF = 0.25.

#### Naïve Bayes

In this section, we will select the training/test method instead of the cross validation, with 10 folds, for the Naïve Bayes classifier and change the percentage of the training samples to study the changes in the classifier accuracy. Table 4 shows the result of these changes.

### Feature extraction

A feature extraction method was performed using Classifier Subset Evaluator by applying a training classification data to estimate the accuracy of these subsets for all used classifiers on the HD dataset and measure the quality of the generated subsets in order to evaluate the classification performance after selecting the relevant attributes per classification algorithm, and the results of the classifier are shown in Table 5, and a visual representation is shown in Fig. 10.

## Discussion

This section discuss the results obtained in the previous section based on the methods used for classification of the HD dataset using different classifiers. Sensitivity Analysis of Decision tree J48 classifier in term of its pruning confidence factor parameter is performed. Then a classifier subset evaluator was used for feature selections of the HD dataset’s features to produce the proposed prediction models for different classifiers.

### Using different classifiers

**Fig. 2:**
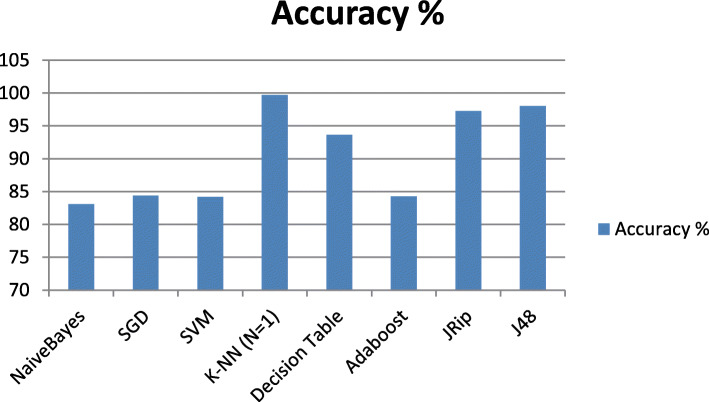
Classification Results in term of the Accuracy

The results seen in Table 2 indicated that using different classification algorithms for the classification of the HD dataset shows very promising results in term of the classification accuracy for the K-NN (K = 1), p.s. all other k values gave similar accuracy, when sensitivity analysis was done on the K-NN classifier, Decision tree J48 and JRip classifiers compared to Naïve Bayes, SGD, SVM, Decision Table and Adaboost classifiers, with accuracy of classification of 99.7073, 98.0488 and 97.2683% respectively, with Kappa statistic value of 0.9941,0.961 and 0.9454 respectively, and it was mentioned earlier, kappa statistics value implies the accuracy of the classification algorithm used as it intent to reach 1, and Fig. 2 shows a graphical representation of the mentioned results.

**Table 2:** Different Classifiers Results

Classifier Used	Accuracy %	kappa	RAE	ROC	MAE	Classification time In seconds
NaiveBayes	83.122	0.6611	39.2	0.902	0.1959	0.02
SGD	84.3902	0.6866	31.24	0.842	0.1561	0.14
SVM	84.1951	0.6825	31.63	0.84	0.158	0.19
K-NN (*N* = 1)	**99.7073**	**0.9941**	**0.69**	**0.994**	**0.0035**	**0.01**
Decision Table	93.6585	0.8734	56.79	0.986	0.2838	0.27
Adaboost	84.2927	0.6857	41.88	0.925	0.2093	0.06
JRip	**97.2683**	**0.9454**	6.31	**0.996**	**0.0315**	0.44
J48	**98.0488**	**0.961**	4.11	**0.996**	**0.0205**	0.27

**Fig. 3:**
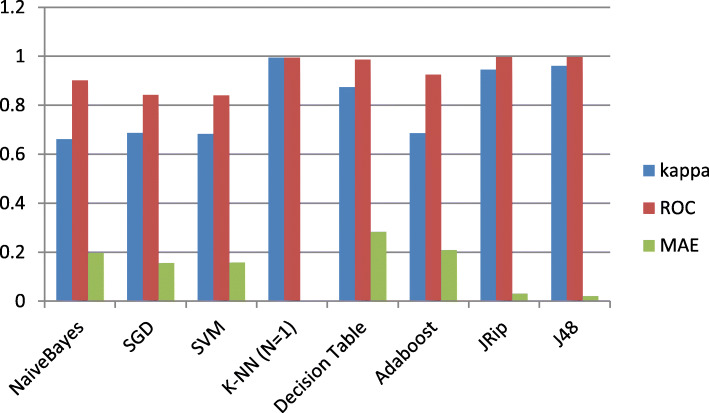
Classification Results in term of the Kappa, ROC and MAE

Figure 3 shows a visual representation of the Kappa statistic, area under curve (ROC) and Mean Absolute Error (MAE) results of the used classifiers presented in Table 2. And we can see the outperformed classification of the K-NN (K = 1), Decision tree J48 and JRip classifiers with respect to the other classifiers with Kappa = 0.9941, ROC = 0.994 and MAE = 0.0035 for the K-NN (K = 1) classifier, Kappa = 0.951, ROC = 0.996 and MAE = 0.0205 for the Decision tree J48 classifier and Kappa = 0.9454, ROC = 0.996 and MAE = 0.0315 for the JRip classifier.

**Fig. 4:**
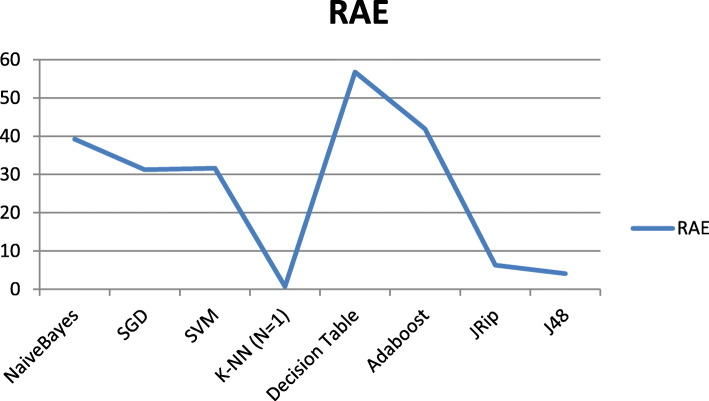
RAE Performance for the Different Classifiers

Figure 4 shows the changes of the Relative Absolute Error (RAE) for the used classifiers to classify the HD dataset, and we can see the K-NN (K = 1) outperform all other classifiers with RAE = 0.69.

#### Confusion matrix

Using the following notations for the confusion matrix for the best classifiers for the classification of the HD dataset: a = 0 value for the class attribute, and b = 1 value for the class attribute.

##### K-NN (k-1)

with 99.7073% accuracy of classified instances.

a b

$$ \left[\begin{array}{cc}499& 0\\ {}3& 523\end{array}\right] $$

##### Decision tree J48

with 98.0488% accuracy of classified instances.

a b

$$ \left[\begin{array}{cc}497& 2\\ {}18& 508\end{array}\right] $$

##### Jrip

with 97.2683% accuracy of classified instances.

a b

$$ \left[\begin{array}{cc}496& 3\\ {}25& 501\end{array}\right] $$

### Parameter’s sensitivity

We will present some parameters sensitivity for Decision tree J48 classifier and change its pruning confidence factor parameter, where smaller pruning value would give more pruning, and we will study the accuracy performance, kappa statistic, MAE and RAE performance of the Decision tree J48 classifier due to these changes.

#### Decision tree J48 pruning confidence factor (PCF)

Table 3 shows the results of the performance of the Decision tree J48 classifier in classifying HD dataset with changes to one of its parameter, PCF, and results show an enhancement in the classification accuracy for the value of PCF = 0.30 and 0.35, where these values are the optimized values for the PCF, with an accuracy of 98.1463% compared to the original results obtained for PCF = 0.25 with 98.0488%. Also, enhancement in for the values of the Kappa statistic = 0.9629, MAE = 0.0189 and RAE = 0.1268 for the PCF = 0.30 and 0.35, compared to Kappa statistic = 0.961, MAE = 0.0205 and RAE = 0.1304 for the value of PCF = 0.25.

**Table 3:** Sensitivity Analysis of the J48 Classifier with Respect to PCF

PCF	Accuracy %	Kappa	MAE	RAE
0.05	96.2927	0.9258	0.0441	0.1099
0.15	97.4637	0.9494	0.0271	0.1501
0.20	97.6585	0.9532	0.025	0.1439
0.25	98.0488	0.961	0.0205	0.1304
**0.30**	**98.1463**	**0.9629**	**0.0189**	**0.1268**
**0.35**	**98.1463**	**0.9629**	**0.0189**	**0.1268**

**Fig. 5:**
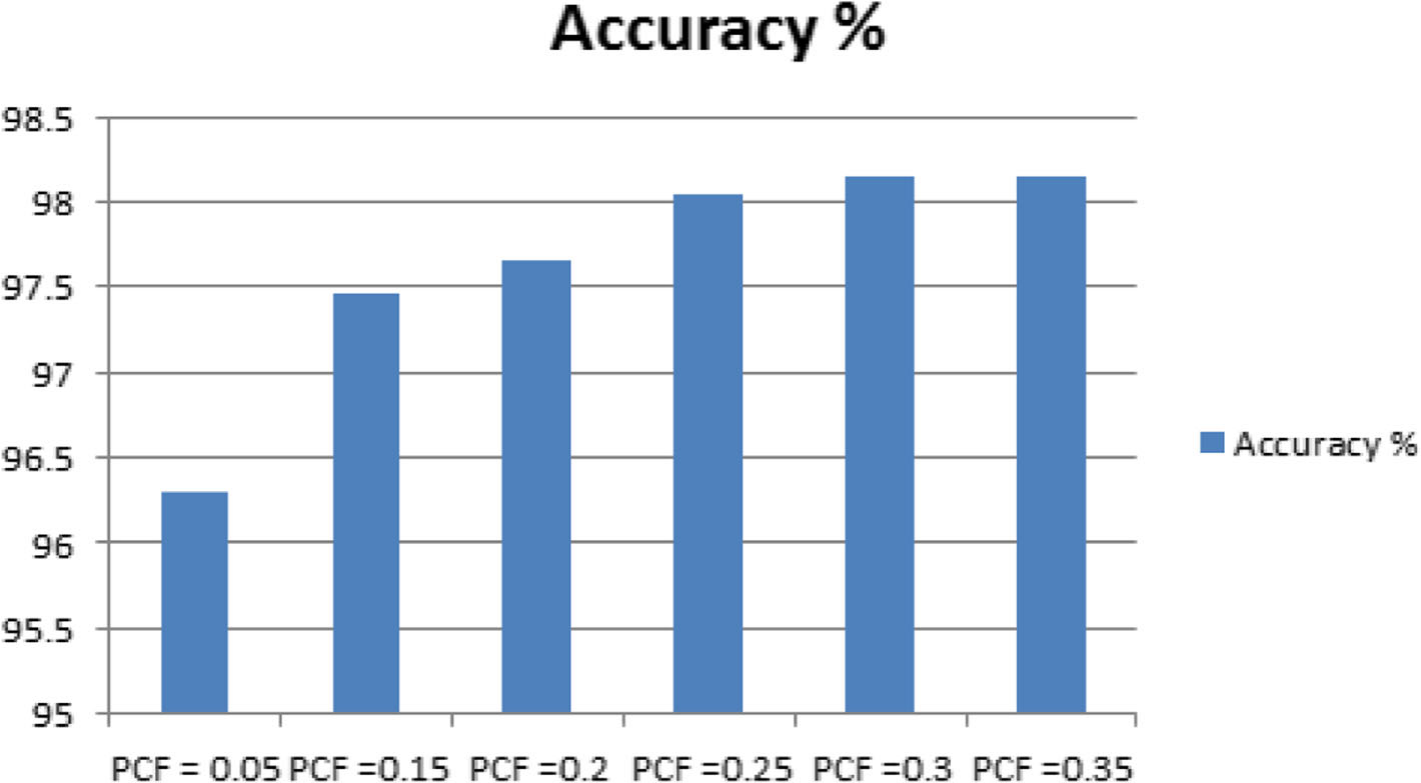
Classification Accuracy Performance with Changes of PCF for J48 Classifier

Figure 5 shows a visual representation of the results obtained in Table 3 for the classification accuracy of the Decision tree J48 classifier for different values of PCF for the classification of the HD dataset.

**Fig. 6:**
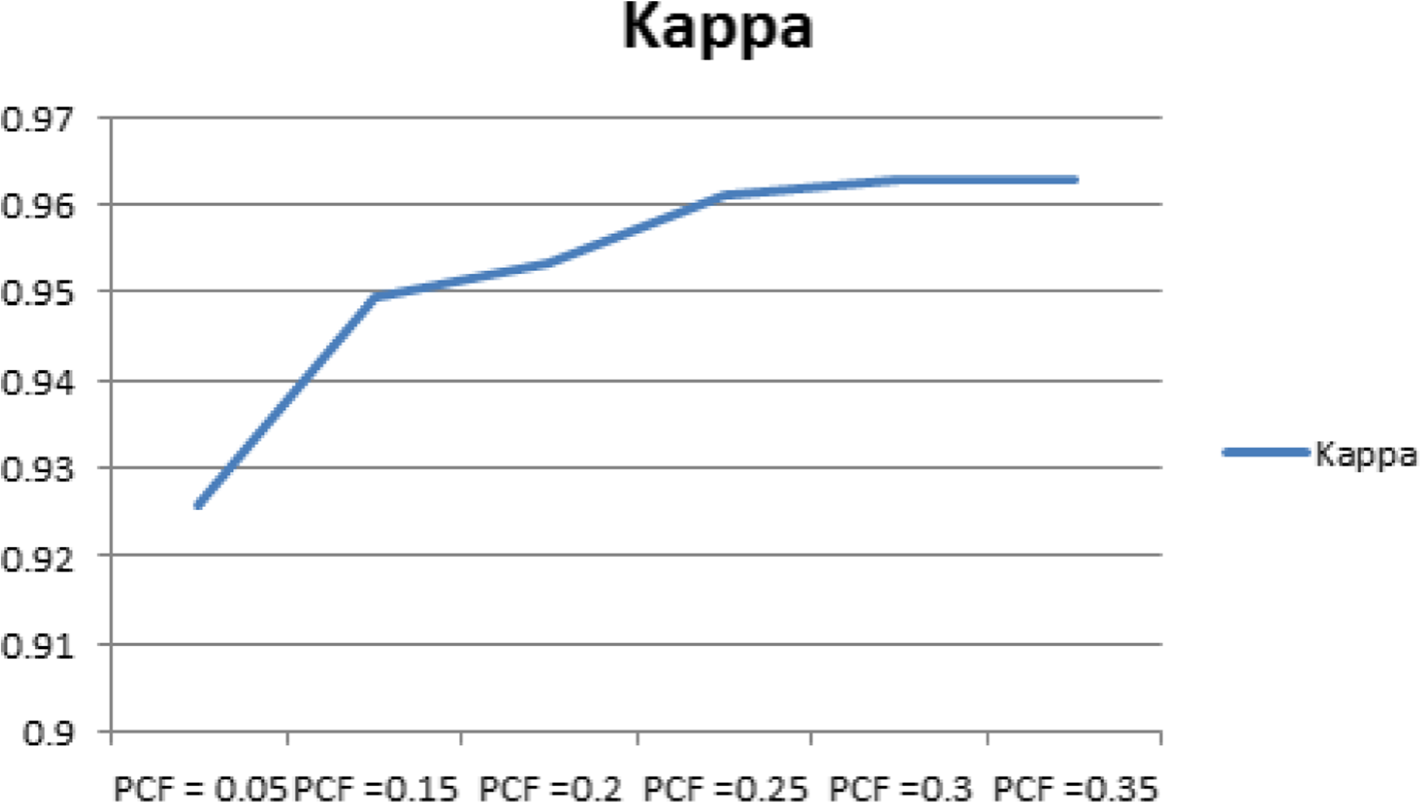
Kappa Statistic Performance with Changes of PCF for J48 Classifier

Figure 6 shows a visual representation of the results obtained in Table 3 for the kappa statistic of the Decision tree J48 classifier for different values of PCF for the classification of the HD dataset.

**Fig. 7:**
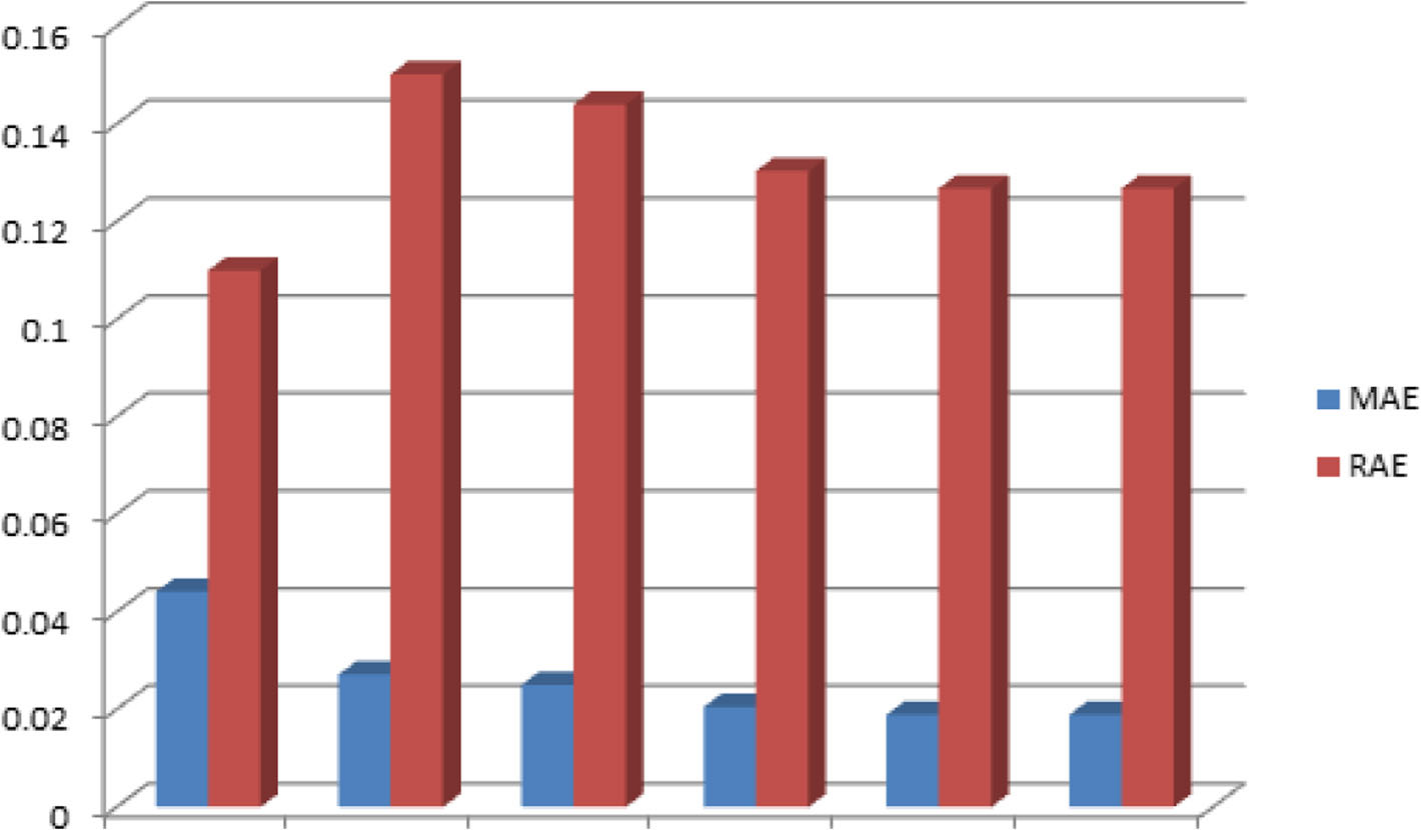
MAE and RAE Performance with Changes of PCF for J48 Classifier

Figure 7 shows a visual representation of the results obtained in Table 3 for the MAE and RAE values for the Decision tree J48 classifier for different values of PCF for the classification of the HD dataset.

#### Naïve Bayes

**Fig. 8:**
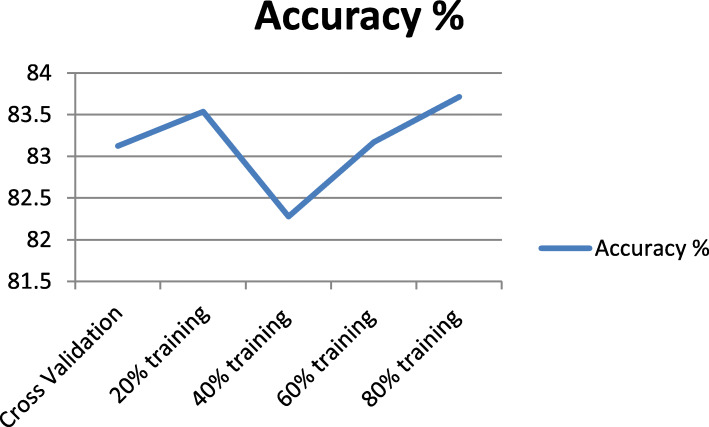
Accuracy Comparison with using Training/Testing Method

We can see from Table 4 the slight changes in the classifier performance in term of classification accuracy, due to the selection of the Training/Testing method instead of the cross validation method. We can see the close performance for accuracy values of 83.5366 and 83.1707% for 20 and 60% training sets respectively compared to the cross validation method with 10 folds, but an outperformance of the Naïve Bayes classifier for the 80% training/testing ratio compared to the cross validation method for an accuracy of 83.7134%. Figure 8 shows an accuracy trend as per the results presented in Table 4.

**Table 4:** Naïve Bays Classifier with Different Training Set

Method used	Accuracy %
Cross Validation	83.122
20% training	83.5366
40% training	82.2764
60% training	83.1707
80% training	**83.7134**

### Feature extraction

**Fig. 9:**
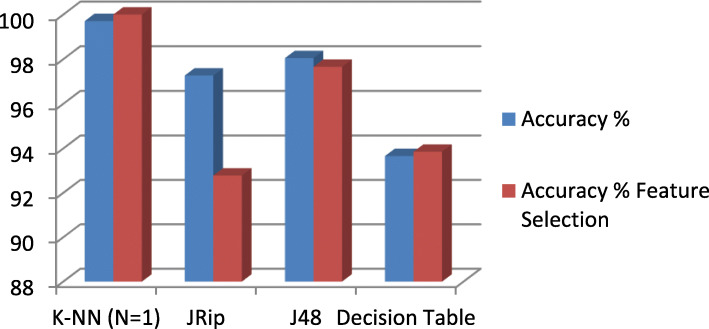
Visual Representation of the Results in Table 5

Table 5 shows the results of the classification algorithms after applying the mentioned feature selection method, and it can be seen that an enhanced performance of increasing of the classifications accuracy for K-NN (*N* = 1) and Decision Table classifiers from 99.7073 and 93.6585% before applying feature selection to 100 and 93.8537% respectively, and a reasonable performance for the Decision tree J48 classifier after feature selection from an accuracy of 98.0488% before feature selection to 97.6585% after feature selection. JRip classifier on the other hand showed a degradation of performance after feature selection. Figure 9 shows a visual representation of the results obtained in Table 5.

**Table 5:** Accuracy Results with Feature Extractions for Different Classifiers for HD Dataset

Features Selected	Accuracy %	Accuracy % Feature Selection	Selected Features
**K-NN (*N*** **= 1)**	99.7073	100	1,5,8
**JRip**	97.2683	92.7805	1,3,5,8
**J48**	98.0488	97.6585	1,3,5,8
**Decision Table**	93.6585	93.8537	1,2,3,5,7,8,9,10,12,13

Table 6 shows the most relevant attributes that can be used for high accuracy classification for K-NN and Decision tree J48 classifiers, in which a very high accuracy of 100% can be obtain to predict a HD case by only applying a combination of up to 4 attributes; consist of age, chest pain type, cholesterol level and maximum heart rate achieved, instead of 13 attributes of the full dataset.

**Table 6:** Extracted Feature per Best Preformed Classifiers

Feature Number	Attribute	Code given	Note
**1**	age	Age	in years
**3**	chest pain type	level of pain	0,1,2,3
**5**	serum cholesterol	cholesterol	in mg/dl
**8**	maximum heart rate achieved	thalach	71–202

## Methods

**Fig. 10:**
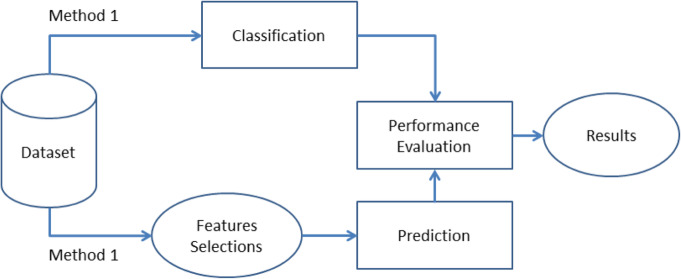
Workflow for the Proposed Methods

In this paper, different mentioned classification algorithms were used to compare these classifiers performance in term of the classification of the mentioned HD dataset, then a feature extraction method was performed using Classifier Subset Evaluator to measure the quality of the generated subsets in order to evaluate the classification performance after selecting the relevant attributes per classification algorithm. Figure 10 shows the workflow for the two used methods.

### Classification methods

We will present number of Machine Learning Classifications algorithm for our analysis [36], in which they will be used for model performance comparison, classification and prediction of HD dataset.

#### K-nearest neighbour (K-NN)

The idea of the Nearest Neighbor (NN) classifier is to take a test data point and comparing it with all training data points and to predict the label (class) of the test data point based on the closest training class using the *L*_*1*_ distance given by:

1 $$ {d}_1\left({I}_1,{I}_2\right)=\sum \limits_p\left|{I}_1^p-{I}_2^p\right| $$

Where *I*_*1*_, *I*_*2*_ are the vectors representation of points *1* and *2* respectively, and *d*_*1*_ denote the distance and ∑ is taken over all points.

#### NaiveBayes

Given the Bayes theorem:

2 $$ P\left(A|B\right)=\frac{P\left(B|A\right)P(A)}{P(B)} $$

For a given elements *A* and *B* and their probability of occurrence *P*(*X*) is calculated, where *P*(*A*) is the probability of occurrence of element *A*, *P*(*B*) is the probability of occurrence of element A and *P*(*A*| *B*) is the conditional probability of element *A* given element *B* occur, and such theorem will be used to perform the classification. So for independent features, the mentioned theorem would perform a direct multiplication of the probability of each feature happening.

#### Decision tree J48

A decision tree model is a model that run number of comparison questions to divide the dataset into different smaller sets based on a given questions (Boolean for instance), and it keeps repeating the task with different set of questions for different level of the available subsets until it covers all available attributes in the dataset. We can have different type of decision tree classifiers based on the nature of the provided questions and their decision rules and based on the nature of the data set. Decision tree J48 is a special case based on the C4.5 algorithm, and it is used for a unified variable associated with the dataset.

#### Decision tree JRip

JRip (RIPPER) is a rule learner classifier found in decision tree algorithm, and uses a repeated incremental pruning for error reduction, and uses four distinctive phases, building, growing, pruning and optimization [37].

#### Stochastic gradient descent (SGD)

Gradient descent is an algorithm that optimizes many loss functions, such as Support Vector Machine (SVM), and Logistic Regression models, and is usually used to optimize the linear function, and the stochastic concept is introduced here based on the roots finding nature of the optimization task. In Stochastic Gradient Descent, for each iteration, samples are selected randomly using a term “batch” for number of samples, instead of the whole data set, and these batches are used to calculate the gradient for each iteration.

#### Support vector machine (SVM)

Given a set of data with N attributes, Support Vector Machine (SVM) classifier is to find a suitable hyper plane in N-Dimensional space that clearly classify the dataset with a maximum margin between data points, where it segregates the two main classes hyper-plane and line to separate the available sets of points, and it is considered a supervised machine learning algorithm which can be used for classification.

#### Adaboost

Is a type of estimator that starts by selecting a set of the original data for fitting on a classifier, and then update the set based on the weight changes of the incorrectly classified instances, until best estimation is achieved [38], and has number of parameters to be considered, such as, base estimator, number of estimators and learning rate.

### Statistical terminologies

The following statistical parameters were used in comparing the evaluation performance of the used classifiers to classify the HD dataset:

#### Relative absolute error (RAE)

RAE Is the value of the relative error divided by the exact value, where the absolute error is the magnitude of the difference between exact value and approximation:

3 $$ RAE=\left|\frac{V_A-{V}_E}{V_E}\right| $$

Where *V*_*A*_ is the approximation and *V*_*E*_ is the exact value respectively.

#### Mean absolute error (MAE)

MAE is a value of the relative error divided by the number of instances, *n*, in a dataset:

4 $$ MAE=\frac{\sum_{i=1}^n\left|{V}_{Ai}-{V}_{Ei}\right|}{n} $$

#### Kappa

Kappa statistic is the value of how close an instance is classified, where the higher Kappa value implies a better classification for a given classifier is performed.

#### Area under curve (ROC)

is a classification parameter to distinguish how well a classifier is [performing in term of the accuracy of identifying data point, and the ideal ROC value for perfect classification is equal to 1.

## Conclusion

In this paper, a comparative analysis of different classifiers was done for the classification of the Heart Disease dataset for positive and negative diagnosed participants. The algorithms were used K- Nearest Neighbor (K-NN), Naive Bayes, Decision tree J48, JRip, SVM, Adaboost, Stochastic Gradient Decent (SGD) and Decision Table (DT) classifiers. It was shown that using different classification algorithms for the classification of the HD dataset produced very promising results in term of the classification accuracy for the K-NN (K = 1), Decision tree J48 and JRip classifiers compared to Naïve Bayes, SGD, SVM, Decision Table and Adaboost classifiers, with accuracy of classification of 99.7073, 98.0488 and 97.2683% respectively, which outperformed other used references in this paper in term of the classification accuracy of 82.5% in [39], 86.43% in [43], 98% in [46], 81.23% in [A], 95% in [D], 93.08% in [G] and 94.62% in [H]. Also results shows Kappa statistic value of 0.9941, 0.961 and 0.9454 respectively. Sensitivity analysis for the Decision tree J48 classifier was applied to study its performance to classify HD dataset with respect to some changes in its pruning confidence factor parameter, and results shows an enhancement in the classification accuracy for the PCF = 0.30 and 0.35, with an accuracy of 98.1463% compared to the original results obtained for PCF = 0.25, and an enhancement in the Kappa statistic, MAE and RAE for the values of 0.9629, 0.0189 and 0.1268 respectively. A feature extraction method was performed using Classifier Subset Evaluator on the HD dataset to evaluate the classification performance after selecting the relevant attributes per classification algorithm. Results show enhanced performance of increasing of the classifications accuracy for K-NN (*N* = 1) and Decision Table classifiers from 99.7073, 93.6585% before applying feature selection to 100 and 93.8537% respectively, compared to 90.40% perdition accuracy in [E], were relevant attributes can be used for high accuracy classification for K-NN and Decision tree J48 classifiers to predict a HD case by only applying a combination of up to 4 attributes instead of 13 attributes of the full dataset. We can clearly see the advantages of this analysis in term of comparing different classifiers to classify the HD dataset, and the benefit of having a reliable feature selection method for HD disease prediction with using minimal number of attributes instead of having to consider all available ones.

## Future work

As an extension to this work, and some sort of limitation to the work performed here, different types of classifiers can be included in the analysis and more in depth sensitivity analysis can be performed on these classifiers, also an extension can be made by applying same analysis to other bioinformatics diseases’ datasets, and see the performance of these classifiers to classify and predict these diseases.

## Data Availability

For the purpose of this study, the Heart Disease dataset provided by. https://www.kaggle.com/johnsmith88/heart-disease-dataset is used, because it is widely used by research community and is publicly available. Creators: 1. Hungarian Institute of Cardiology. Budapest: Andras Janosi, M.D. 2. University Hospital, Zurich, Switzerland: William Steinbrunn, M.D. 3. University Hospital, Basel, Switzerland: Matthias Pfisterer, M.D. 4. V.A. Medical Center, Long Beach and Cleveland Clinic Foundation:Robert Detrano, M.D., Ph.D.

## Abbreviations

HD: Heart disease
SGD: Stochastic Gradient Decent
DT: Decision Table
SVM: Support Vector Machine
ML: Machine Learning
K-NN: K- Nearest Neighbor
CAD: Coronary Artery Disease
HDPS: Heart Diseases Prediction System
NN: Neural Networks
CAS: Carotid artery stenting
DWT: Discrete wavelet transform
1D-HLP: 1-dimensional hexadecimal local pattern
RAE: Relative absolute error
MAE: Mean Absolute Error
ROC: Area Under Curve
PCF: Pruning Confidence Factor
